# Relative Hypodense Vertebral Artery Sign on Computerized Tomography in Atherosclerotic Near Occlusion

**DOI:** 10.1155/2016/3506161

**Published:** 2016-12-29

**Authors:** Muhammad Faraz Raghib, Slaven Pikija

**Affiliations:** ^1^Aga Khan University, Medical College, Karachi, Pakistan; ^2^Department of Neurology, Christian Doppler Medical Centre, Paracelsus Medical University Salzburg, Salzburg, Austria

## Abstract

A 52-year-old white male presented with an acute onset of slurred speech along with hypoesthesia in the entire left arm. The acute computed tomography (CT) showed relative hypodensity in the intracranial segment of left vertebral artery (VA) that was not present in historical images, pointing to the possible lack of flow. The site of occlusion was confirmed by magnetic resonance imaging (MRI) that showed susceptibility effect in the affected artery. By means of historical native CT comparison the site of VA thrombosis was correctly predicted. Local atherosclerotic thrombosis of the VA could be relatively hypodense on native CT and still have positive susceptibility weighted imaging (SWI) sign.

## 1. Introduction 

Ischemic strokes etiologies are categorized as large-artery atherosclerotic, small vessel occlusion, cardioembolic, undetermined, and other special causes or unknown [1]. Large-artery atherothrombotic infarctions involve local thrombotic occlusions mostly as the consequence of sudden rupture of underlying atherosclerotic plaque in the carotid, vertebrobasilar, and intracranial arteries. Approximately 85% of all strokes are ischemic, and 20% of them occur in the vertebrobasilar system. MRI is the method of choice since it is significantly more sensitive than CT in detection of ischemic lesion within 48 hours of stroke onset (88% versus 72%, *P* = 0.01) [2]. The vessel imaging plays a crucial role in planning further therapies.

## 2. Case Presentation

Here we describe a case of a 52-year-old white male with a history of transitory ischemic attack in the vertebrobasilar territory, arterial hypertension, hyperlipidemia, and mild chronic kidney disease. He presented to us with slurred speech. The National Institutes of Health score is 2. The symptoms started 23 hours before presentation with an acute onset of slurred speech along with hypoesthesia in the entire left arm. Four months ago, he was hospitalized because of transitory paresis and hypoesthesia in the entire right arm and leg that lasted for 1 hour. Brain MRI showed right intracranial VA stenosis and he was started on aspirin which he takes regularly.

**Figure 1:**
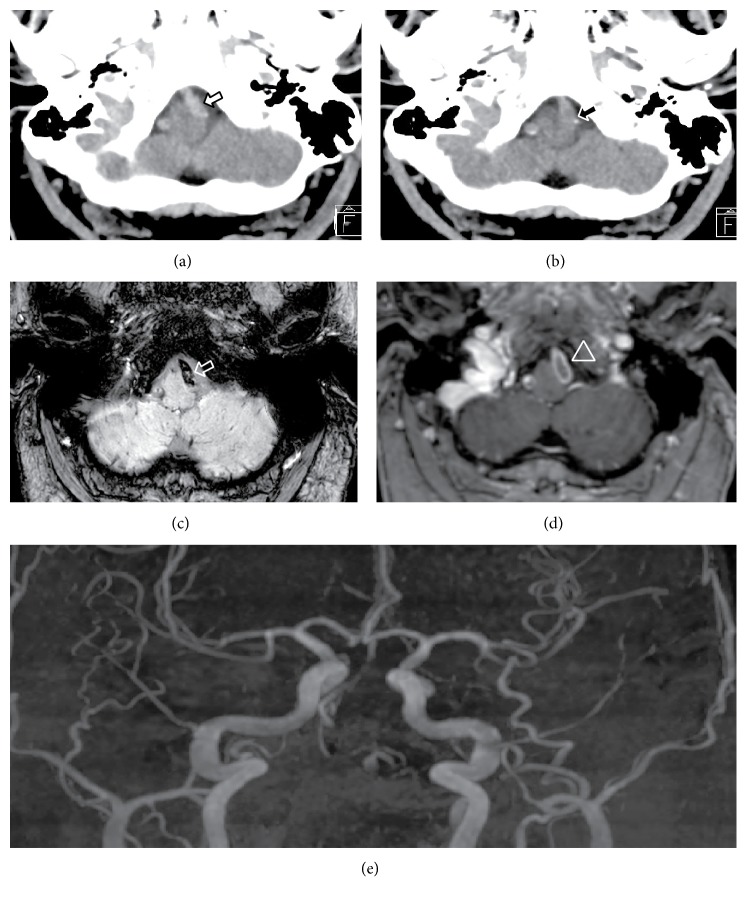
Seven months before presentation left VA is patent with intraluminal average Hounsfield units (aHU) of 60.6 on native CT (open black arrow) (a). 23 hours after symptom onset. Black arrow shows subtle hypodensity with aHU of 42.4 in the left VA (closed white arrow) (b). Three days after symptom onset susceptibility weighted effect in left VA (empty white arrow) (c) and flow void with rest-flow around clot in contrast-enhanced MR T1 (open white arrowhead) (d). MR time-of-flight angiography showing the absence of flow in both VAs (e).

The sensory symptoms spontaneously remitted after 60 minutes. Creatinine is 1.23 mg/dL (0.67–1.17) and C-reactive protein is 1.24 mg/dL (0.00–0.50). The MRI showed fresh ischemic lesions in the area of right middle cerebellar peduncle and right crus cerebri, the areas served by anterior inferior cerebellar and posterior cerebral artery, respectively. The acute CT showed relative hypodensity in the intracranial segment of left VA and MRI showed susceptibility effect in the same and in contralateral VA. MR angiography showed incomplete occlusion of both VAs while the basilar artery is without thrombotic formations (Figure 1).

The etiology is presumed to be of local atherosclerotic origin and he was started on dual antiplatelet therapy and made a good recovery with modified Rankin scale of 2 on discharge.

## 3. Discussion

The eye of the stroke neurologist is trained to recognize arterial hyperdensities, so the arterial flow voids are easily overseen on native CT. The presence of hyperdense artery sign on native CT is highly specific (95%) albeit not sensitive (52%) for the arterial occlusion with thin slices improving its detection [3]. The presence of significant hypodense middle cerebral artery sign is mostly linked to fat embolus [4]. SWI is 83% sensitive and up to 100% specific for acute middle cerebral artery occlusion [5, 6]. SWI is sensitive to hemoglobin degradation products. SWI sign is thought to be associated mostly with cardioembolic clots since red blood cells are major component of it [7, 8]. SWI sign in VA thrombosis is seldom reported and correlation with etiology is not known [9]. However data are scarce for vertebral artery regarding both techniques. In some centers there is lack of access to MRI and up to 10% acute patients MRI is contraindicated for various reasons ranging from severe claustrophobia to heart pacemaker to nonfixed metal parts in head. For the subset of patients the CT could be the only viable option. In our patient, given the dramatic progression of the occlusive VAs process in only 7 months we believe that rapid expansion and consequently the rupture of the plaque surface produced distal embolisation.

We are showing that in special circumstances the native CT when examined in detail correlated with clinical symptoms and compared to historical images could lead to correct vascular diagnosis.
